# Hungarian general practice paediatricians’ antibiotic prescribing behaviour for suspected respiratory tract infections: a qualitative study

**DOI:** 10.1136/bmjopen-2023-081574

**Published:** 2024-05-10

**Authors:** Balázs Babarczy, Ágnes Hajdu, Ria Benko, Mária Matuz, Renáta Papp, Pantelis Antoniou, Ketevan Kandelaki, Danilo Lo Fo Wong, Sahil Khan Warsi

**Affiliations:** 1 Syreon Research Institute, Budapest, Hungary; 2 National Center for Public Health and Pharmacy, Budapest, Hungary; 3 Department of Clinical Pharmacy, University of Szeged, Szeged, Hungary; 4 Centre of Science & Innovation Vice-rector and Business Development, Semmelweis University, Budapest, Hungary; 5 WHO Regional Office for Europe, Copenhagen, Denmark

**Keywords:** Health policy, Public health, GENERAL MEDICINE (see Internal Medicine)

## Abstract

**Abstract:**

**Objectives:**

Inappropriate antibiotic prescribing is a major cause of antimicrobial resistance (AMR). The aim of this study was to explore paediatric general practitioners’ (GP Peds) antibiotic prescription practice in suspected respiratory tract infections (RTIs), using the capability–opportunity–motivation–behaviour framework.

**Design:**

The design is a qualitative study based on individual, semistructured telephone or virtual interviews.

**Setting:**

Paediatric general practice in Hungary. We applied stratified maximum variation sampling to cover the categories of age, sex and geographical location of participants.

**Participants:**

We interviewed 22 GP Peds. Nine were male and 13 were female: 2 of them were less than 40 years old, 14 were between 40 and 60 years, and 6 were above 60 years. 10 worked in low-antibiotic prescription areas, 5 in areas with medium levels of antibiotic prescription, 3 in high-antibiotic prescription areas, and 4 in and around the capital city.

**Results:**

Study participants had varying antibiotic prescription preferences. Personal experience and physical examination play a central role in GP Peds’ diagnostic and treatment practice. Participants emphasised the need to treat children in their entirety, taking their personal medical record, social background and sometimes parents’ preferences into account, besides the acute clinical manifestation of RTI. Most respondents were confident they apply the most effective therapy even if, in some cases, this meant prescribing medicines with a higher chance of contributing to the development of AMR. Some participants felt antibiotic prescription frequency has decreased in recent years.

**Conclusions:**

Our findings suggest that a more prudent attitude toward antibiotic prescribing may have become more common but also highlight relevant gaps in both physicians’ and public knowledge of antibiotics and AMR. To reinforce awareness and close remaining gaps, Hungary should adopt its national AMR National Action Plan and further increase its efforts towards active professional communication and feedback for primary care physicians.

**STRENGTHS AND LIMITATIONS OF THIS STUDY**

- The study followed a rigorous qualitative methodology anchored in behaviour change theory.
- Our stratified maximum variance sampling strategy assured breadth in terms of representing different situations.
- The relatively small sample size precludes generalisability.
- As participation was voluntary, there could be possible selection bias where participants choosing to participate were already aware of and interested in antimicrobial resistance.

## Background

 The misuse of antibiotics, including unnecessary or suboptimal treatment, can cause patient harm. In addition, inappropriate antibiotic use is a leading cause of development of antimicrobial resistance (AMR).^1^ Known as the silent pandemic, AMR represents 1 of the 10 major global threats to humanity today. AMR endangers antibiotics’ therapeutic benefit and imposes an avoidable cost burden on healthcare systems.^2^

While Hungary has developed, but is still to adopt its National Action Plan on AMR, recommended by the WHO, an evidence brief for policy on antibiotic misuse in Hungary identified multiple contributing factors including inappropriate prescribing practices, public misconceptions, prescribing pressure from patients, unavailability of certain narrow-spectrum antibiotics, healthcare system resource deficiencies and marketing by pharmaceutical companies.^3^ One policy solution advanced in the brief is increasing the awareness of health practitioners and the general public about rational antibiotic use and AMR. This is in line with the priorities of the WHO Global Action Plan on AMR.^4^

Analysis of Hungarian health insurance data on publicly reimbursed antibiotics (circa 95% of the total dispensed) shows children as the highest consumers of antibiotics, mostly prescribed broad-spectrum antibiotics. Children are an important patient group, as they often suffer from viral infections, particularly viral respiratory tract infections (RTIs) not requiring antibiotic therapy. More than 70% of community antibiotic prescriptions in Hungary are written by general practitioners (GPs). GPs in Hungary work in three types of practices: adult-only, mixed (adult–paediatric) and paediatric practices. Adult-only and mixed practices are served by physicians with general practice or related specialisations (eg, internal medicine). On the other hand, general practice paediatricians (GP Peds) specialise in paediatrics before starting to work in dedicated paediatric practices. Adult practice GPs have the highest share of all antibiotic prescriptions in the country (35%), but GP Peds and GPs in mixed adult and paediatric practices prescribe the most antibiotics per doctor. Antibiotic dispensing data analysis details are presented in online supplemental file 1.^5^

In light of the antibiotic prescribing data and policy brief findings, research was conducted on Hungarian GP Peds’ prescribing behaviour. The WHO Regional Office for Europe supported this pilot study under the Tailoring Antimicrobial Resistance Programmes (TAP) process, developed to support Member States in developing interventions to address the spread of AMR.

## Study goals and objectives

The study aimed to identify factors affecting GP Peds’ behaviour of prescribing antibiotics for suspected RTIs and drivers and barriers to appropriate prescribing.

Research objectives were to collect data on GP Peds’:

1. Diagnostic process for suspected RTIs.
2. Decision-making on antibiotic prescribing.
3. Choice of active agent prescribed.
4. Views on appropriate interventions to improve antibiotic prescription behaviour.

This article reports results for the first three objectives related directly to antibiotic prescribing behaviour.

Following the TAP approach, research was built on the Behaviour Change Wheel (BCW) model to develop public health interventions to target identified health behaviour barriers and drivers. BCW is grounded in the capability–opportunity–motivation–behaviour (COM-B) theory,^6^ which holds that individuals’ performance of specific public health behaviours is influenced by three inter-related factors: capability, availability and motivation. Capability refers to individuals’ knowledge or skill to perform the behaviour, such as a GP’s awareness of antibiotic resistance. Opportunity addresses contextual factors external to the individual, such as the availability of narrow-spectrum antibiotics or social attitudes toward antibiotics. Motivation concerns individuals’ decision-making on behaviour performance, such as a GP’s consideration of workload, patient demands and personal attitudes.

## Methods

### Sampling and recruitment

Existing data showed GP Peds’ prescription habits differed across groups. A maximum variation, stratified sampling strategy^7^ was thus used to increase the diversity of responses. Quotas were set to aim for equal distribution by strata of sex and geographical location, and distribution by age to reflect national statistics of GP Peds’ prescription ratios. All GP practices in Hungary are funded by the National Health Insurance Fund, which randomly sampled GP Peds in their contracting database from lists created according to the strata mentioned above. As privacy protection rules precluded access to data on age, the year of graduation was used as a proxy, assuming the average age of graduation as being 24 years.

The randomly selected GPs were contacted by researchers using a publicly available contact database. Non-respondents were replaced with a similar-profile GP from the list. Those agreeing to participate were contacted directly by the interviewers via telephone or email to make an appointment for an interview.

### Data collection

Data were collected via semistructured interviews. The interview guide (online supplemental file 2) was based on a review of guides from other qualitative studies on antibiotic prescribing/use.^8^,^16^ The WHO Regional Office for Europe supported interview guide development and researcher training on conducting qualitative data collection and analysis. Interview guides explored factors influencing GP Peds’ prescribing, namely: their knowledge and experience; external actors, such as parents, peers, pharmaceutical sales representatives or public policy; and their personal considerations. Interviews were conducted in Hungarian, tape-recorded and transcribed verbatim. Due to the COVID-19 pandemic, interviews were conducted via telephone or other virtual platforms instead of face-to-face.

### Data analysis

This study was conducted in accordance with the Consolidated criteria for Reporting Qualitative research statement^17^ (online supplemental file 3). Transcripts were analysed using a deductive framework approach to identify emerging themes.^18 19^ Data in the form of participants’ words, statements and ideas were analysed to recognise themes relating to GP Peds’ prescribing behaviour. Data were analysed by five researchers, using a coding framework that was developed based on an initial sample of transcripts, and continuously updated through a consensus. Coded transcript segments were copied into a shared document, organised by themes derived from the COM-B theory, and subheadings were developed through a consensus based on emerging themes. Each finding was represented through exemplary quotations taken from participant responses.

Following initial thematic data analysis, a second COM-B analysis was conducted to consider findings on barriers and drivers to GP Peds’ prescribing behaviour. One researcher reviewed initial findings and identified COM-B themes in the data. Results were verified and refined by researchers who conducted data collection and initial analysis.

## Results

### Study population

22 GP Ped interviews were conducted between July and December 2021. The participant breakdown is presented in table 1. Original strata quotas could not be exactly met due to participant refusals (n=20). Female participants were in a slight majority (59.1%). The majority of participants were mid-career doctors (63.6%), which did not deviate significantly from the envisioned 55% quota. The number of early-career doctors interviewed (n=2) was below the envisioned target of 15%. The maximum variation sample was originally composed to represent even distribution among low-prescription, medium-prescription and high-prescription counties, and the capital region of Budapest and Pest County. However, low-prescription counties were over-represented (n=10, 45.5%) in the final sample compared with high-prescription counties (n=3, 13.6%).

**Table 1:** Participant breakdown by sex, age and geography

Variable	Number of participants
Sex	
Male	9
Female	13
Year of graduation (approximate age group)	
After 2005 (<40 years)	2
Between 1985 and 2005 (40–60 years)	14
Before 1985 (>60 years)	6
Geography	
Low-prescription counties	10
Medium-prescription counties	5
High-prescription counties	3
Budapest and Pest County	4

### Findings on prescribing behaviour

GP Peds’ antibiotic prescription behaviour was analysed thematically around: (1) the diagnostic process to assess the presence of a bacterial infection; (2) the choice of when to prescribe or not to prescribe antibiotics; and (3) the choice of which antibiotic agent to use. There was a high level of diversity in prescription behaviour among participants. Some patterns were identified, but did not always correlate with participants’ age, sex or geographical location. The main thematic findings on behaviour are summarised in table 2.

**Table 2:** Summary of main themes relative to prescription behaviour

Diagnostic process	Physical examination is key.Laboratory blood testing and microbiological testing are occasional, depending on the clinical situation.Point-of-care testing is present in a few practices with some having given it up.
When to prescribe antibiotics	Most claim prescribing very few antibiotics, and some emphasise prescription has decreased over the years.Most claim not to prescribe antibiotics for viral infections, and some mention specific symptoms precluding prescribing.Some refer children to diagnostics or recommend symptomatic treatment instead.The majority wait a couple of days from symptom onset before prescribing antibiotics.Some issue delayed prescriptions to parents in specific cases.
Choice of antibiotic agent	Most claim using narrow-spectrum penicillins, extended-spectrum penicillin combinations or mainly second-generation cephalosporins followed by macrolides, according to the type and severity of symptoms.There is individual variation in the relative importance of the different groups of agents prescribed.Patient-specific considerations of child’s age, frequency of administration and other factors may also influence the decision on the antibiotic prescribed.

#### Diagnostic process

Diagnostics are key to accurately determine disease aetiology (ie, viral infections or bacterial infections indicating the need for antibiotic use). Physical examination took precedence for all participants and was almost the only method of diagnosis for some, especially older GP Peds relying on decades-long clinical experience.

I examine [the children], listen to them, look at them, and put that couple of years’ experience in it. (late-career male doctor, Budapest/Pest County)

OK, there are these [point-of-care] tests for laboratory examination, but I don’t have such a machine, but with 41-years experience, I look at [the child’s] tonsil, and … can estimate if it is *Streptococcus*, *Haemophilus*, or another infection. (late-career male doctor, high-prescription county)

In a minority of cases, physical examination was complemented with laboratory blood testing and/or microbiological testing. Laboratory blood testing was mainly used for targeted mononucleosis diagnosis, but some participants used it for more general purposes, such as C reactive protein testing or long-lasting infections diagnosis. Microbiological testing was mentioned less frequently. Some respondents used it for pathogen determination. Point-of-care testing (POCT) was also rare. Several participants indicated the availability of, and having used, POCT equipment in the past, but having given it up or seldomly using it due to a lack of time or financial reasons. In the case of suspected community-acquired pneumonia, some respondents referred to ambulatory X-ray examination, while others preferred direct reference to a hospital ward.

#### Decision to prescribe antibiotics

All participants claimed not to prescribe antibiotics for presumably viral infections, with some also specifying when they refrain from prescribing. Several claimed prescribing antibiotics only on few occasions, and some emphasised that their antibiotic prescribing had decreased in recent years. Some mentioned specific symptoms considered in deciding on antibiotic prescription, though symptoms listed differed between respondents.

I don’t immediately start an antibiotic when I don’t see patches, and only a red, swollen tonsil. Sinusitis is excluded below five and upper respiratory tract infections are mostly viral, so I give a lot of consideration to whether an antibiotic is needed or not. (mid-career female doctor, low-prescription county)

…for tonsillitis follicularis, we usually give an antibiotic without delay… If we’ve heard something [in the respiratory tract], then we almost always prescribe antibiotics. (late-career male doctor, medium-prescription county)

Almost all participants said they waited a couple of days from symptom onset before prescribing antibiotics, allowing for an immune system response to infection. Some referred children to diagnostics or recommend symptomatic treatment. Some also issue delayed prescriptions to parents in specific cases.

[for] lengthy viral infection… parents can tolerate [waiting], so let’s have a blood test first… and we can manage 2 days for results [without antibiotics]. (mid-career male doctor, medium-prescription county)

#### Choice of antibiotic agent

Most participants decided on an antibiotic agent considering the type and severity of symptoms, generally starting with narrow-spectrum penicillin, followed by extended-spectrum penicillin combinations, cephalosporins (mainly second and sometimes third generation), and finally macrolides and other antibiotics. There was individual variation in importance ascribed to these groups of antibiotics.

For tonsillitis, we always start with [narrow-spectrum] penicillin… If the penicillin was not initially appropriate, or then for otitis, I usually choose amoxicillin-clavulanic acid or cefixime… These last years, in cases [of atypical pathogens], I prefer [medicines] containing azithromycin taken once a day, and if [children] get on well, a 3-day course may be enough… (early-career female doctor, low-prescription county).

Some GP Peds, especially in low-prescription counties, reported using narrow-spectrum penicillin often, while others started treatment with amoxicillin or amoxicillin–clavulanic acid combinations. Some GPs were open to using cephalosporins, while others said they used them less compared with when first introduced on the market. While most respondents only used macrolides for atypical infections, some reported using them more frequently. Respondents also indicated the decision on active agents could be influenced by factors such as the child’s age or frequency of administration, which could influence compliance.

### Findings on COM-B factors affecting prescribing behaviour

Findings presented some potential COM-B-related barriers and drivers to appropriate prescribing behaviour.

#### Capability

Participants’ belief that their experience grants them sufficient expertise to diagnose and treat RTIs could both be a driver and a barrier. This confidence could be leveraged to encourage appropriate prescribing but could also indicate knowledge gaps on AMR and in prescribing guidelines. It should be noted that clinical protocols for outpatient antibiotic treatment exist for eight common types of infection in Hungary. However, these protocols are not officially promulgated, so some practitioners rely on international guidelines instead. Participants’ widely varying attitudes toward different antibiotics could also indicate gaps in knowledge and/or use of existing guidelines. Regarding sources of information, participants reported receiving information via conferences, continuing education, professional newsletters and websites, and outreach from medical sales representatives, which reinforced their existing perceptions.

The knowledge I have is completely sufficient, so I don’t feel a need for anything [like additional education or training]. (late-career female doctor, medium-prescription county)

I read these guidelines, but to be honest, the last one I read was about 5 years ago. (mid-career male doctor, low-prescription county)

[Amoxicillin] is recommended in the [national] protocols, and sometimes I also consult international guidelines… [like] the European Paediatric Association… [and an] English paediatric website, I don’t remember its name. (mid-career female doctor, Budapest/Pest County)

#### Opportunity

A significant finding related to opportunity concerns barriers to using laboratory blood testing, microbiological testing and POCT. Respondents indicated they were not used frequently due to the long turnaround times and, in the case of POCT, the lack of time during appointments and lack of reimbursement. A social opportunity barrier was identified in the form of social attitudes to antibiotics and compliance with doctors’ recommendations. Participants indicated parents could influence prescription behaviour based on economic grounds, such as not having sick leave, or because of a lack of awareness or education. However, several respondents noted a post-pandemic trend following the period of telemedicine, with parental attitudes moving away from antibiotics and the majority of parents accepting doctors’ recommendations.

There is… [a] social class for whom it’s difficult to explain that the child can heal even after 1 week, and that it can be a viral infection. But otherwise even with them, with time… one can make progress. (early-career female doctor in a low-prescription county)

Parents today don’t like unnecessary antibiotic prescription. They even condemn the doctor who immediately prescribes an antibiotic, saying they don’t like it, they don’t agree with it. There’s no more pressure to give antibiotics like there was, say, 15 years ago. (mid-career female doctor, low-prescription county)

#### Motivation

Participants’ responses presented two motivation-related findings. First, respondents are focused on effective treatment and make risk-averse decisions to avoid complications or ineffective treatment in the short term. In this case, awareness of the problem of AMR was reported by some as a reason to prescribe wide-spectrum antibiotics to avoid treatment failure. This barrier is related to a knowledge gap, where patient outcomes’ connection to AMR can seem distant and difficult to grasp. Second, participants make prescription decisions based on personal views of symptoms and antibiotics, taking into account the individual nature of each patient (eg, potential consequences regarding side effects, deterioration of the microbiome, tastes).

I don’t really prescribe simple penicillin. I also don’t give, like some guidelines recommend, a small dose of amoxicillin first, and then if [the infection] doesn’t respond to that, give a higher [dose]. I find this quite risky… With pneumonia, after 3 days I increase, and after 6 days I have therapeutic failure. I don’t really see the point in this. We know that some Pneumococci only respond to a higher dose, so I give a normal [dose of] beta-lactam, and then I know there won’t be such a problem. (mid-career male doctor, medium-prescription county)

## Discussion

### Consideration on findings

The study’s findings on GP Peds’ antibiotic prescribing for suspected RTIs revealed that practitioners rely mainly on physical examination of the patient for making diagnosis, with mid-career and late-career doctors having high confidence in this method, based on their experience. Despite availability, fewer participants reported using laboratory blood testing, microbiological testing or POCT, though early-career doctors were more likely to use targeted blood and microbiological testing. The lack of time is a contextual barrier that was identified, contributing to the lack of uptake of available testing options, which could suggest a need for further training or adjustments to practices to facilitate and encourage testing use. In other recent studies, time and patient pressure were similarly found to impact prescribing behaviour, particularly by favouring more frequent prescribing of broad-spectrum antibiotics.^20 21^

Most participants indicated prescribing very few antibiotics, prescribing less over the last few years and not prescribing antibiotics for viral infections. A decreasing trend can also be seen in national data for antibiotic consumption in the community (primary and secondary outpatient care combined), but the rate of this was moderate until the COVID-19 pandemic.^22^ Some studies suggest that public health awareness campaigns, clinical guidelines and continuing education resources for clinicians can help combat overprescribing among early-career physicians, especially in cases of conflicting prescribing practices and advice from more senior colleagues.^23^ Additionally, while the COVID-19 pandemic has led to an increase in the use of broad-spectrum antibiotics in some cases, research shows that, in some primary care settings, antimicrobial consumption decreased during the first wave in multiple countries.^24 25^ Similar trends of a decrease in antibiotic use at the community level have been reported in Hungary.^26^ Risk avoidance and diagnostic uncertainty have been noted as important themes for the prescription of antibiotics in multiple cases in several countries,^21^ which is in line with early-career doctors’ tendency to rely on diagnostic testing compared with mid-career and late-career doctors who reported high confidence in their diagnostic capabilities.

The decision to prescribe antibiotics was reportedly made several days after symptoms appear, though variation was found in the symptoms for which respondents would prescribe, as well as in the choice of antibiotic prescribed. Many participants made prescribing decisions based on their perceptions of the children’s context, potential compliance and consideration of the short-term effectiveness of treatment. These findings could suggest barriers in the form of knowledge gaps, and a risk-averse approach to patient treatment, focusing on a patient’s short-term outcomes, as observed in other contexts.^27 28^ Potential ways to address such barriers include training and education on appropriate prescribing, balancing longer-term AMR and shorter-term effectiveness considerations.^29^

### Strengths and weaknesses of the study

This study fills an important gap, as no prior qualitative research on the determinants of prescribing behaviour among a high-prescribing population has been conducted in Hungary. One strength of the study is the use of rigorous qualitative methodology in following behaviour change theory to inform study design and data collection and analysis.

While our stratified maximum variance sampling strategy assured breadth in terms of different situations represented, the relatively small sample size precludes generalisability. However, the robust adherence to theory-based methodology ensures that results express relevant dynamics affecting practitioners’ behaviour, which raises further questions for enquiry relevant to policy decisions. As participation was voluntary, there could be a possible selection bias with participants who are already aware of and interested in AMR choosing to participate. An additional limitation is that research had been planned prior to, but conducted during, the global COVID-19 pandemic.

We cannot assert that thematic saturation was achieved; hence, there is a possibility of additional themes that were not identified in this study. Another limitation is that research participants were not given the opportunity to provide feedback on the conclusions of this paper, which raises the possibility of misinterpretation of some of their statements. However, a separate and more detailed research report in Hungarian will be disseminated among the research participants for their comments. Patients and/or the public were not involved in the design, or conduct, or reporting, or dissemination plans of this research.

## Conclusion

Hungarian GP Peds participating in this qualitative study demonstrated varying antibiotic prescription preferences for children with RTI. Their knowledge about antibiotics and the treatment of infections stems from diverse sources, but personal experience has a key role. They also reported different levels of access to diagnostic tools, including laboratory blood testing, microbiological testing and POCT, but physical examination was the key element of their diagnostic approach, in any case. They put great emphasis on treating children in their entirety, taking into account their personal medical record, social background and, sometimes, their preferences. Most of them are also confident that they apply the most effective therapy, despite eventual pressure from parents. Importantly, several doctors have the impression that antibiotic prescription is less frequent than it used to be and that parents themselves are less likely to ask for it. These findings reflect positive trends in the attitudes of both physicians and the general population, but also reveal gaps in knowledge and opportunity. To reinforce this trend and close remaining gaps, in accordance with the WHO Global Action Plan on AMR, Hungary should adopt its National Action Plan on AMR, publish official guidelines on antibiotic use, and further increase its efforts towards active professional communication and feedback for primary care physicians.

## supplementary material

10.1136/bmjopen-2023-081574 online supplemental file 1

10.1136/bmjopen-2023-081574 online supplemental file 2

10.1136/bmjopen-2023-081574 online supplemental file 3

10.1136/bmjopen-2023-081574 online supplemental file 4

## Data Availability

No data are available.
