# Supplementary material for: Hungarian general practice paediatricians’ antibiotic prescribing behaviour for suspected respiratory tract infections: a qualitative study
Source: BMJ Open. 2024 May 10;14(5):e081574. doi: 10.1136/bmjopen-2023-081574 (PMC11097800; doi:10.1136/bmjopen-2023-081574)
Supplement: online supplemental file 1 [file bmjopen-14-5-s001.pdf]

Supplementary file 1: Analysis of national antibiotic dispensing data – main results

We have analysed data on antibiotic (ATC: J01) dispenses obtained from the National Health Insurance Fund administration for 2016-2017. The following variables were included in the analysis:

- Specialty of prescriber (general practice, ear-nose-throat, pulmonology, urology, obstetrics-gynaecology, other)
- Age group of prescriber (3 groups)
- Sex and age group of patient
- Geography of patient's residence (county and type of settlement).

The output variables included the number of prescriptions, the units of drugs dispensed and consumption in daily defined doses (DDDs).

A summary of results can be accessed here: https://spark.adobe.com/page/TTBqteuAzkCKL/.
