# Supplementary material for: Hungarian general practice paediatricians’ antibiotic prescribing behaviour for suspected respiratory tract infections: a qualitative study
Source: BMJ Open. 2024 May 10;14(5):e081574. doi: 10.1136/bmjopen-2023-081574 (PMC11097800; doi:10.1136/bmjopen-2023-081574)
Supplement: online supplemental file 2 [file bmjopen-14-5-s002.pdf]

Supplementary file 2: Interview guide

Introduction

Objectives: To introduce the interviewer and the interviewee to each other; to inform on research scope/purpose; and to sign consent forms.
Est. duration: 5-10 minutes.

- Introductions
- Asking permission for the interview to be recoded, and turning the device on if the answer is positive
- Introduction to research scope and purpose (time allowed for questions if any)
- Signing the consent form if the interview is in person, or asking for short verbal confirmation on the terms of the consent form signed and sent back via email, in case of telephone/online interviews

Notes: Make sure that the informants have read the consent form in advance of the interview. (In case it was not done before the interview, time should be allowed for it now.) Bring a few copies along, plus pens. For telephone interviews, consent forms should be sent via email in advance.

Exploration

Objective: To explore in-depth what influences the target group in performing perceived roles and responsibilities, through use of the COM factors: capability, opportunity and motivation.

Est. duration: 30-40 minutes.

Notes: Expect overlap in responses, so keep flexibility. Go along with the conversation and focus on primary questions where relevant – depth is important in this section. Some categories may not be relevant to a specific informant/group, so if the responses are limited, move on. Make sure informants fully understand what is being asked.

Research question	Lead sentence	Primary questions	Secondary questions
1. **How is it decided whether an AB is prescribed for an RTI to child?**	Please think of one or two recent cases when you prescribed AB for an RTI to a child.	• What did you think through before prescribing an AB?<br>• How did you diagnose whether it was a viral or bacterial infection?<br>• What diagnostic examinations/tests you carried out or ordered, and why these?<br>• Special circumstances, e.g.:<br>- COVID-19<br>- borderline case<br>- approaching weekend/travel<br>- socio-economic factors	• Describe the consultations – how did they go? (intro) Did the consultations take place in person or online due to COVID-19?<br>• How did you come to the diagnosis? (C) What was your rationale for diagnosing this way?<br>• What symptoms make you think an RTI is viral or bacterial? What diagnostic tools, you use?<br>• How did patient factors (symptoms, symptom duration, status, other conditions) play a role in choosing treatment? (C/M)<br>• How would you describe your patient population in terms of socio-economic background, health literacy, preference for in person vs online consultation etc.?<br>• How did patient/parent preferences/pressure play a role in your treatment decisions? How did you recognise their expectations? (O/M)<br>• How did the quality of doctor-patient/parent relationship play a role? (O)<br>• How did access to different tests (blood test, microbiological test, point-of-care test), time pressure or financing play a role in your diagnostic and/or treatment decisions? (O)<br>• Which guidelines played a role? (O)<br>• How did information transmitted by pharmaceutical representatives or at events sponsored by pharmaceutical companies play a role? (M)<br>• Were these one/two recent cases typical for your practise?<br>• Were there any specific cases that resulted in changes in your general prescription practise of ABs? (M)<br>• What would you need to become more confident in your diagnoses? (C/O)
2. **How is it decided which active agent is prescribed for an RTI to child?**	Please think of one of the cases we have been talking about so far when you decided to prescribe an AB.	• How did you decide which specific agent to use?<br>• What was the AB of your choice and why?	• Which antibiotic product did you prescribe? Why did you choose this specific product?<br>• How did AB features (taste, dosage frequency, storage, side effects etc.) play a role? (M)<br>• How do patient/parent preferences play a role? How did you know about their expectations? (O/M)<br>• How did information transmitted by pharmaceutical representatives or at events sponsored by pharmaceutical companies play a role? (M) How often do you

Research question	Lead sentence	Primary questions	Secondary questions
			receive information on specific ABs from pharmaceutical companies? Do you find information obtained from companies trustworthy?<br>• Any guideline played a role? (C/O)<br>• How did the availability of antibiotics in pharmacies play a role? (O)<br>• How did the price of ABs for the patient play a role? (O)<br>• Were there any previous situations (related to a specific AB) that changed your antibiotic preferences (M)? If so, could you describe those?<br>• Could you please list the most frequently prescribed ABs you prescribed for RTI during the last year? Why do you prefer these? (Prompts: broad vs narrow spectrum, dosage, recommended by guidelines or pharmaceutical representatives)
3. **In which cases or circumstances does no prescribing take place?**	Please think of the last one or two cases when you did NOT prescribe AB for an RTI.	What are the reasons when you decide not to prescribe AB for an RTI?	• How did the patient/parent react when you had decided not to prescribe an AB? (M)<br>• Was it necessary to provide a detailed explanation to the parent? If so, what was your explanation?<br>• How did your communication skills help you in the situation? Do you find it easy or challenging to handle such situations? (C)<br>• How did the quality of doctor-patient/parent relationship play a role, if any? (O)<br>• Did you experience any pressure (by parent, supervisor, company, authority) to prescribe an antibiotic for RTI? (O/M) If yes, can you please provide example(s)? How do you recognise their expectations? In what ways do you feel pressured? Do you sometimes feel that you have to give in to the pressure?<br>• Were there any situations when deciding not to prescribe ABs resulted in a negative outcome? (M)
4. **Sources of information on ABs**	Please summarize how do you obtain information on ABs and your typical sources of information on ABs	• What are the sources that you find more trustworthy than others?<br>• Why do you find them more trustworthy?	• What information channels do you use to get information on ABs? (e.g. journals, courses, conferences, congresses, webinars, pharmaceutical representatives) (O, C) How often do you seek information on ABs? (M)<br>• Why do you find these sources/channels useful/effective? (C)<br>• Where do you think patients/parents get information about their health?<br>• Do you find professional courses, continuing medical education relevant in your practice? (C)

Research question	Lead sentence	Primary questions	Secondary questions
			• Have you attended any training courses on/including AB prescribing in the last 2 years? (O, M) If so, which professional body/company was the organiser? Were you satisfied with the information obtained and its practicality?<br>• If you have not attended such a course recently, what was the reason? (e.g. lack of interest, high costs, lack of substituting GP, poor access or no knowledge about such courses)<br>• Which factors could support the participation of pediatric GPs at training courses on ABs?<br>• What types of information do you receive from pharmaceutical representatives? How useful are these in your practice?
5. **Antibiotic resistance (ABR)**	We would now touch upon ABR given that it is associated with antibiotic use	• Have you ever encountered an infection caused by an antibiotic-resistant pathogen in your practice?<br>• Is ABR a problem in your practice?<br>• How do you know whether it is a problem or not?	• How big a problem do you think ABR is in Hungary?<br>• Where do you get professional information on ABR?<br>• Do you think your patients/parent are aware of the ABR problem? Do you address this topic when communicating with patients/parents in your practice?<br>• Where do you think patients/parents get information about ABR?
6. **What interventions can work in the Hungarian GP context, according to the GP peds opinion?**	We hope that this research can help pediatric GPs in areas important to them in prescribing ABs	What would you propose as a policy or intervention which could help maintain appropriate prescribing and reduce inappropriate AB use in general practice in Hungary?	• Why do you think these would be effective?<br>• How do you think GP peds would react to this? What would be the challenges of implementation?<br>• What do you think would be most applicable to your situation?<br>• Would you appreciate guidelines or decision algorithms? Would it be appropriate if they were mandatory?<br>• How helpful would it be for you to communicate your decisions on AB prescribing if there were short leaflets that could be given to the patient/parent?<br>• What are your views on interventions targeting patients/parents? How could we increase their knowledge and awareness?

Research question	Lead sentence	Primary questions	Secondary questions
			• Have you heard about the European Antibiotic Awareness Day (ECDC) / World Antibiotic Awareness Week (WHO) held on 18 November each year?<br>• What types of messages and communication channels would you prioritise in a possible Hungarian campaign?
7. **National Health Insurance Fund (NEAK) quality indicators**	The practice gets 1 score in an 8-score system if the mean number of AB dispenses are below the county average	• What is your opinion on the NEAK quality indicator on ABs?<br>• How useful it is in your opinion?<br>• How relevant it is in your opinion?	

Closure

Objectives: To ensure that informants and researchers have the opportunity for final comments or questions; to inform on how informants can contact researcher if they have further questions/comments; and to make sure that the interview is properly closed.

Est. duration: 5-10 minutes.

- Final perspectives and comments from the informant (if any)
- Final questions from researchers (if any)
- Confirming contact details of researchers and expected timeline for sharing findings
- Thanks and goodbye
