# Supplementary material for: Hungarian general practice paediatricians’ antibiotic prescribing behaviour for suspected respiratory tract infections: a qualitative study
Source: BMJ Open. 2024 May 10;14(5):e081574. doi: 10.1136/bmjopen-2023-081574 (PMC11097800; doi:10.1136/bmjopen-2023-081574)
Supplement: online supplemental file 3 [file bmjopen-14-5-s003.pdf]

Supplementary file 3: Consolidated criteria for reporting qualitative studies (COREQ) checklist

**Domain 1: Research team and reflexivity**	
Personal characteristics	
Interviewer/facilitator	BR, MM, BB, HA
Credentials	Dr. Pharm, PhD (BR, MM), economist, PhD (BB), Dr. Med (HA)
Occupation	Pharmacologist (BR, MM), health data analyst (BB), public health specialist (HA)
Gender	Female (BR, MM, HA), Male (BB)
Experience and training	Trained through multiple workshops focusing on theoretical and practical aspects of both the Tailoring Antimicrobial Resistance Projects (TAP) methodology and qualitative methodology, organised by the World Health Organization Regional Office for Europe.
Relationship with participants	
Relationship established	No
Participant knowledge of the interviewer	No
Interviewer characteristics	This was not addressed.
**Domain 2: Study design**	
Theoretical framework	
Methodological orientation and Theory	Thematic content analysis mapped to the COM-B framework
Participant selection	
Sampling	Random sampling with maximum variation taking into account age, sex and region
Method of approach	Telephone invitation
Sample size	22 in total
Non-participation	20 individuals (18 refused to participate, 2 agreed to participate, but dropped out before the interview). Reasons: lack of time, being busy, seeing no point in this research
Description of sample	Age (2 participants under 40, 14 between 40 and 60 years of age, and 6 over 60);<br>Sex (9 male, 13 female participants);<br>Region (10 participants from counties with low prescription rate, 5 from counties with medium prescription rate, 3 from counties with high prescription rate, 4 from Budapest [capital city] and Pest county)
Setting	
Setting of data collection	21 telephone interviews, 1 interview through web-based

	video call (The locations of the participants at the time of the interviews - e.g. GP office, home - were not documented.)
Presence of non-participants	No
Data collection	
Interview guide	Interview guide drafted, piloted and revised. Additional questions have been added due to the COVID-19 pandemic.
Repeat interviews	None
Audio/visual recording	Interviews were audio recorded
Field notes	Ad-hoc note taking during the interviews
Duration	40-70 minutes, an hour on average
Data saturation	Sampling until data saturation had been planned, but eventually only 20 participants were enrolled due to feasibility reasons
Transcripts returned	Transcripts were not returned to participants for comment and/or correction
**Domain 3: analysis and findings**	
Data analysis	
Number of data coders	5 in total
Description of the coding tree	A coding tree was not developed, themes were mapped to COM-B
Derivation of themes	Both themes identified in advance and derived from the data were mapped and analysed
Software	No qualitative data analysis software was applied. Microsoft Word was used to manage the data.
Participant checking	This was not conducted
Reporting	
Quotations presented	Participant quotations are extensively presented to illustrate the themes/findings. Each quotation is identified by participant number.
Data and findings consistent	Yes
Clarity of major themes	Major themes are clearly presented in the findings.
Clarity of minor themes	Diverse cases and discussion of minor themes are presented in detail.
