# Supplementary material for: Hungarian general practice paediatricians’ antibiotic prescribing behaviour for suspected respiratory tract infections: a qualitative study
Source: BMJ Open. 2024 May 10;14(5):e081574. doi: 10.1136/bmjopen-2023-081574 (PMC11097800; doi:10.1136/bmjopen-2023-081574)
Supplement: online supplemental file 4 [file bmjopen-14-5-s004.pdf]

# Supplementary file 4: Informed Consent Form

**University of Szeged**
**Department of Clinical Pharmacy**
Head of Department [redacted]

**Szegedi Tudományegyetem Klinikai**
**Gyógyszerészeti Intézet**
Intézetvezető: [redacted]

office.clph@pharm.u-szeged.hu
6725 Szeged, Szikra u. 8.
Hungary
tel/fax: 62/544-921

## Informed Consent Form for General Practice Paediatricians

This informed consent form is for general practice paediatricians in Hungary, whom we are inviting to participate in the Hungarian study of the European Tailoring Antimicrobial Resistance Programmes project, titled "Qualitative analysis of Hungarian general practice (GP) paediatricians' antibiotic prescribing capability, opportunity, motivation and behaviour".

**Principle Investigator:** [redacted]
**Organization:** University of Szeged, Department of Clinical Pharmacy
**Sponsor:** World Health Organization Regional Office for Europe
**Project:** Tailoring Antimicrobial Resistance Programmes (TAP) Hungary

**This Informed Consent Form has two parts:**

- **Information Sheet (to share information about the study with you)**
- **Certificate of Consent (for signatures if you choose to participate)**

**You will be given a copy of the full Informed Consent Form.**

## Part I: Information Sheet

### Introduction

This informed consent form has been developed for research within the World Health Organization's (WHO) Tailoring Antimicrobial Resistance Programmes (TAP) initiative in Hungary. We are kindly asking you to take part in this research, which comprises of an interview at your GP office, at another location you deem appropriate or over the telephone/online or internet-provided platform.

**Purpose of the research**

Antimicrobial resistance is a major challenge for healthcare on the global level today, and antibiotic use is one of its key drivers. With this research project, we are striving to underpin future policy interventions to support clinicians, particularly GPs, in Hungary and across the WHO European Region in improving their antibiotic prescription practices and so advancing the combat against antibiotic resistance.

**Type of Research Intervention**

Within this research, we conduct semi-structured interviews. A semi-structured interview is a slightly directed conversation between me and you, which will last around 60-90 minutes.

**Participant Selection**

The reason that we are seeking to understand the patterns and motivations of antibiotic use in paediatric general practice is that this sector has a particular impact on community antibiotic prescription, according to our analysis of national data on medicine use provided by the National Health Insurance Fund.

**Voluntary Participation**

Participation is entirely voluntary. You decide whether you would like to take part or not. You may also stop participating in the interview any time you wish. You may change your mind later and stop participating even if you agreed earlier. In case you declined to take part at the interview, there will be no negative consequences for you, now or at any later stage.

**Procedures**

I will ask you some questions about your choices and experiences regarding antibiotic prescription to children with respiratory tract infections. There are no right or expected answers to these questions: our objective is to understand, to the best possible extent, the situation in which your decisions are made and the factors that you take into account. The interview, if you agree to this, will be audio tape recorded, and will then be transcribed and analysed. The recording will be deleted immediately after the transcript has been made. If you do not agree to have our interview audio-recorded, now or at any later stage of the interview, our interview can still continue and I will take notes of your answers instead.

**Duration**

The research takes place over ten months in total. We will make interviews with 20 general practitioner paediatricians altogether.

**Risks**

We have not identified any risks associated with taking part in this evaluation. However, if you may feel uncomfortable talking about some of the topics, you do not have to answer any question or take part in the interview if you do not wish to do so. You do not have to give us any reason for not responding to any question, or for refusing to take part in the interview. None of this will have negative consequence on you or your practice, we will respect your choices.

**Benefits**

This study is conducted to support the development of policy interventions. Better knowledge about the reasons of your antibiotic choices could help us design interventions that could increase the spread of evidence-based practice in community care.

**Reimbursements**

You will not be provided any monetary incentive to take part in the research.

**Confidentiality**

Participation in this research is anonymous. Any information you share will be confidential and cannot be traced back to you when results and findings are summarized. The transcript of this interview will be anonymised and stored at a secure drive of the University of Szeged. It will only be accessible to members of the research working group, for the strict purposes of the research study.

**Sharing the Results**

Nothing that you tell us today will be shared with anybody outside the research team, and nothing will be attributed to you by name. The knowledge that we get from this research will be summarized in a final report. It will be shared with you for comments before we present it to WHO, the Ministry of Human Capacities, the National Public Health Center and the National Healthcare Service Center, to be used for the development of targeted interventions. Scientific publications about the study results are also foreseen afterwards.

**Right to Refuse or Withdraw**

I will give you an opportunity at the end of the interview to review your remarks, and you can ask to modify or remove portions of those.

**Who to Contact**

With any questions you should have, you may contact any of the following people:

**Principal investigator, University of Szeged, Department of Clinical Pharmacy** [redacted], assistant professor, [redacted]

**Technical supervision, WHO**: [redacted] technical officer, [redacted]

**This proposal has been reviewed and approved by the Scientific and Research Ethics Committee of the Health Science Council, which is a committee whose task it is to make sure that research participants are protected from harm. If you wish to find about more about the committee, contact its secretary, [redacted] at [redacted], or at [redacted]. It has also been reviewed by the Ethics Review Committee of the World Health Organization (WHO), which is sponsoring and supporting the study.**

You can ask me any more questions about any part of the research study, if you wish to. Do you have any questions?

**Part II: Certificate of Consent**

**I have been invited to participate in a qualitative study which aims to explore the reasons of antibiotic use and antibiotic choice in pediatric general practice. I have read the foregoing information, and I consent voluntarily to be a participant in this study.**

**Print Name of Participant__________________**

**Signature of Participant ___________________**

**Date ___________________________**

**Day/month/year**

**Statement by the researcher/person taking consent**

**I have accurately read out the information sheet to the potential participant, and to the best of my ability made sure that the participant understands that the following will be done:**

1. **a semi-structured interview will be conducted;**
2. **it will be tape-recorded;**
3. **anonymity will be assured.**

**I confirm that the participant was given an opportunity to ask questions about the study, and all the questions asked by the participant have been answered correctly and to the best of my ability. I confirm that the individual has not been coerced into giving consent, and the consent has been given freely and voluntarily.**

**A copy of this ICF has been provided to the participant.**

**Print Name of Researcher/person taking the consent________________________**

**Signature of Researcher /person taking the consent__________________________**

**Date ___________________________**

Day/month/year
