# Supplementary material for: Hungarian general practice paediatricians’ antibiotic prescribing behaviour for suspected respiratory tract infections: a qualitative study
Source: BMJ Open. 2024 May 10;14(5):e081574. doi: 10.1136/bmjopen-2023-081574 (PMC11097800; doi:10.1136/bmjopen-2023-081574)
Supplement: Supplementary file 5 [file bmjopen-2023-081574.reviewer_comments.pdf]

PEER REVIEW HISTORY

BMJ Open publishes all reviews undertaken for accepted manuscripts. Reviewers are asked to complete a checklist review form (**http://bmjopen.bmj.com/site/about/resources/checklist.pdf**) and are provided with free text boxes to elaborate on their assessment. These free text comments are reproduced below.

ARTICLE DETAILS

**TITLE (PROVISIONAL)**	Hungarian general practice paediatricians' antibiotic prescribing behaviour for suspected respiratory tract infections – a qualitative study
**AUTHORS**	Babarczy, Balázs; Hajdu, Ágnes; Benko, Ria; Matuz, Mária; Papp, Renáta; Antoniou, Pantelis; Kandelaki, Ketevan; Lo Fo Wong, Danilo; Warsi, Sahil Khan

VERSION 1 – REVIEW

**REVIEWER**	Davey, Andrew<br>University of Newcastle School of Medicine and Public Health
**REVIEW RETURNED**	29-Nov-2023

**GENERAL COMMENTS**	The subject of the research is important.<br>The research question is clearly stated.<br>The methodology used is appropriate to the research question.<br>Despite the limitations encountered when conducting the study there is sufficient rigour to accept the findings.<br><br>Minor editing and suggestions<br>Background<br>It would be useful to have a brief definition of GP Peds to help understand their role and training for international audiences. In other health systems 'paediatrician' is a word that suggests a specialisation in child-only internal medicine and not as a general practitioner. I am not familiar with the Hungarian health system, but I am assuming that GP Peds are doctors who trained as specialist general practitioners in primary care who decide to sub-specialise in child-only primary care.<br><br>Methods<br>Page 8 around line 47 – Who or what is the Regional Office?<br><br>Strengths and Weaknesses<br>The following two methodological issues should be described because they reduce the trustworthiness (credibility) of the findings:<br>• thematic saturation was not reached hence there is a possibility of further themes that were not identified,<br>• there was no participant checking which leaves the reader uncertain that the participants would have agreed with the interpretation of their responses.<br><br>Patient and Public Involvement<br>Page 19 around line 46 to 47 – 'board' should be 'broad'

**REVIEWER**	Ivanovska, V<br>WHO
**REVIEW RETURNED**	26-Dec-2023

**GENERAL COMMENTS**	Please clarify if Hungary has adopted its National Action Plan (NAP) on AMR. The article says that Hungary needs to adopt its national AMR strategy and action plan, so please clarify if at least any (form of) NAP on AMR has been developed. If not (which can be surprising), this shall be the priority recommendation for action. Related to this, be specific if Hungary has developed antibiotic guidelines about infections and related syndromes' management (i.e. diagnosis and treatment), and if so, when, and how have they been communicated to prescribers, and GP compliance monitored. (And can you make any recommendations about restricting the outreach from medical sales representatives or introducing the antimicrobial stewardship concept in PHC?) All of my comments above could hopefully be considered under the relevant COMB factors, and addressed in the background or in the discussion/recommendation sections. One update to the narrow spectrum vs broad spectrum antibiotic discussion is to introduce the AWaRe classification in this manuscript as this is now being used as a specific EU strategy to assess the appropriateness of use of antibiotics.) It is good for the context that the authors mentioned the unavailability of some antibiotics and the need for sharing antibiotic prescribing data with the GPs.<br><br>Also improve the conclusions of the abstract by using a more concise technical language - e.g. favourable trends in the attitudes toward antibiotic use - favourable is not clear. And be more specific with the recommendations as suggested above.<br><br>Well done and good luck!

**REVIEWER**	Csonka, Peter<br>Tampere University, Tampere Center for Child, Adolescent and Maternal Health Research, Faculty of Medicine and Health Technology
**REVIEW RETURNED**	05-Jan-2024

**GENERAL COMMENTS**	Overall, I find the study to be commendable for its well-designed methodology, novelty, and clear presentation. The authors have successfully addressed an important knowledge gap in Hungary providing valuable insights into potential problems involving antibiotic use in the country.<br><br>The manuscript is well-written. The authors effectively communicate complex concepts in a clear and concise manner, making the content accessible to a broad readership. The use of appropriate terminology and the organization of the manuscript contribute to its overall readability.<br><br>The methodology section is thorough and well-structured, providing a clear overview of the experimental design and data collection procedures. The study demonstrates a sufficient level of scientific design, execution, and analysis. The sample size is small and limits the power of the data, but it has been discussed sufficiently. I consider this study to be a explorations for further projects, especially to develop meaningful actions plans.

	The results section clearly presented. The use of tables and figures is judicious, aiding in the interpretation of findings. The authors have succeeded in providing a detailed account of both positive and negative aspects of the study, contributing to the transparency of the study.<br><br>The conclusions drawn by the authors are supported by the data presented, but will need further studies to have a greater impact on appropriate antibiotic use in the community.

VERSION 1 – AUTHOR RESPONSE

Reviewer 1
Background
It would be useful to have a brief definition of GP Peds to help understand their role and training for international audiences. In other health systems 'paediatrician' is a word that suggests a specialisation in child-only internal medicine and not as a general practitioner. I am not familiar with the Hungarian health system, but I am assuming that GP Peds are doctors who trained as specialist general practitioners in primary care who decide to sub-specialise in child-only primary care. – We have added a brief description at page 4 of the manuscript.

Methods
Page 8 around line 47 – Who or what is the Regional Office? – WHO Regional Office for Europe. Has been clarified in the text.

Strengths and Weaknesses
The following two methodological issues should be described because they reduce the trustworthiness (credibility) of the findings:
• thematic saturation was not reached hence there is a possibility of further themes that were not identified,
• there was no participant checking which leaves the reader uncertain that the participants would have agreed with the interpretation of their responses.
We have added a paragraph at page 12, describing both potential issues.

Patient and Public Involvement
Page 19 around line 46 to 47 – 'board' should be 'broad' – Corrected.

Reviewer 2
Please clarify if Hungary has adopted its National Action Plan (NAP) on AMR. The article says that Hungary needs to adopt its national AMR strategy and action plan, so please clarify if at least any (form of) NAP on AMR has been developed. If not (which can be surprising), this shall be the priority recommendation for action. – The country has prepared, but not yet adopted its NAP. We have specified this at page 4, and also highlighted it in the conclusions (page 13).
Related to this, be specific if Hungary has developed antibiotic guidelines about infections and related syndromes' management (i.e. diagnosis and treatment), and if so, when, and how have they been communicated to prescribers, and GP compliance monitored. - Clinical protocols for outpatient antibiotic treatment have been developed and published, but not officially promulgated. We have specified this at page 10, and included the publication of guidelines among our suggestions in the conclusion (page 13).
(And can you make any recommendations about restricting the outreach from medical sales representatives or introducing the antimicrobial stewardship concept in PHC?) – The developed NAP

addresses these challenges as well. Therefore, in our view, the first priority would be its adoption and subsequent implementation.
One update to the narrow spectrum vs broad spectrum antibiotic discussion is to introduce the AWaRe classification in this manuscript as this is now being used as a specific EU strategy to assess the appropriateness of use of antibiotics. It is good for the context that the authors mentioned the unavailability of some antibiotics and the need for sharing antibiotic prescribing data with the GPs. – Unavailability of certain narrow-spectrum antibiotics in Hungary is an issue known to experts, but it was not underlined as an important factor by our interviewees. Therefore, we could not build specific recommendations about this here. However, those are present in the draft NAP.

Also improve the conclusions of the abstract by using a more concise technical language - e.g. favourable trends in the attitudes toward antibiotic use - favourable is not clear. And be more specific with the recommendations as suggested above. – We have edited the abstract and the conclusions accordingly.

Reviewer 3
Overall, I find the study to be commendable for its well-designed methodology, novelty, and clear presentation. The authors have successfully addressed an important knowledge gap in Hungary providing valuable insights into potential problems involving antibiotic use in the country.

The manuscript is well-written. The authors effectively communicate complex concepts in a clear and concise manner, making the content accessible to a broad readership. The use of appropriate terminology and the organization of the manuscript contribute to its overall readability.

The methodology section is thorough and well-structured, providing a clear overview of the experimental design and data collection procedures. The study demonstrates a sufficient level of scientific design, execution, and analysis. The sample size is small and limits the power of the data, but it has been discussed sufficiently. I consider this study to be a explorations for further projects, especially to develop meaningful actions plans.

The results section clearly presented. The use of tables and figures is judicious, aiding in the interpretation of findings. The authors have succeeded in providing a detailed account of both positive and negative aspects of the study, contributing to the transparency of the study.

The conclusions drawn by the authors are supported by the data presented, but will need further studies to have a greater impact on appropriate antibiotic use in the community.

Comments do not require further action.

VERSION 2 – REVIEW

**REVIEWER**	Davey, Andrew<br>University of Newcastle School of Medicine and Public Health
**REVIEW RETURNED**	27-Feb-2024

**GENERAL COMMENTS**	Well done.
